# Secular trends and correlates of physical activity: The Tromsø Study 1979-2008

**DOI:** 10.1186/s12889-016-3886-z

**Published:** 2016-12-03

**Authors:** Bente Morseth, Bjarne K. Jacobsen, Nina Emaus, Tom Wilsgaard, Lone Jørgensen

**Affiliations:** 1 Department of Community Medicine, UiT The Arctic University of Norway, Tromsø, Norway; 2 School of Sport Sciences, UiT The Arctic University of Norway, Tromsø, Norway; 3 Centre for Clinical Research and Education, University Hospital of North Norway Trust, Tromsø, Norway; 4 Department of Health and Care Sciences, UiT The Arctic University of Norway, Tromsø, Norway; 5 Department of Clinical Therapeutic Services, University Hospital of North Norway, Tromsø, Norway

**Keywords:** Exercise, Leisure time physical activity, Occupational physical activity, Secular trends, Correlates, Prevalence

## Abstract

**Background:**

The aim was to describe secular trends in leisure time physical activity (LTPA) and work related physical activity (WPA) from 1979 to 2008. Additionally, we explored potential cross-sectional and longitudinal correlates of LTPA and WPA.

**Methods:**

Data are collected from 34,898 individuals (49.7% men) aged >20 years who participated in at least one Tromsø Study survey between 1979 and 2008. In each survey, the participants completed a self-administered questionnaire and underwent physical examinations. LTPA and WPA were assessed by the validated “Saltin-Grimby” 4-scale questions. Potential correlates of LTPA and WPA (sex, age, body mass index (BMI), education, smoking, self-reported cardiovascular disease, self-perceived health, and employment status) were tested using ordinal logistic regression.

**Results:**

The age-adjusted prevalence of participants being inactive in leisure time remained relatively stable around 20% from 1979 to 2008 (range 19.9–23.6%). The age-adjusted prevalence of moderate-vigorous LTPA decreased from 23.2% in 1979–80 to 16.0% in 2001, thereafter the prevalence increased to 24.3% in 2007–08 (*P* <0.05). The age-adjusted prevalence of being mostly sedentary at work increased gradually from 35.5% in 1979–80 to 53.4% in 2007–08 (*P* <0.05). Sex, age, education, and smoking were identified as cross-sectional correlates of LTPA and WPA (*P* <0.05). Men had higher odds of engaging in LTPA than women (adjusted OR 1.52 [95% CI 1.39–1.67] in 2007–08), whereas the association between sex and WPA shifted over time. High education level, not being a smoker, and high WPA were associated with high LTPA, whereas low education level, being a smoker, and high levels of LTPA were associated with high WPA (*P* <0.05). In general, odds of engaging in LTPA and WPA decreased with age (*P* <0.05). Individuals with healthy BMI had higher odds of being in a higher LTPA level than those who were underweight and obese (*P* <0.05). Longitudinal analyses identified sex, age, education, smoking, WPA, and LTPA measured in 1979–80 as determinants of LTPA in 2007–08.

**Conclusions:**

In Norwegian adults, the proportion of sedentary WPA increased from 1979 to 2008, whereas the proportion of inactive LTPA remained stable. Being female, older, smoker, obese or underweight, and low education level were associated with low LTPA levels.

**Electronic supplementary material:**

The online version of this article (doi:10.1186/s12889-016-3886-z) contains supplementary material, which is available to authorized users.

## Background

Over the last decades, mechanization and technological advances have reduced the need for bodily movement in order to perform daily tasks. These societal changes would be expected to be reflected in a decline in total physical activity level in the population. However, a review of world-wide secular trends indicates that leisure time physical activity (LTPA) has actually increased over time [1], and recent studies support this increasing trend in LTPA over the last decades [2–9]. For example, the proportion of physically active men during leisure time increased by almost 10% in Canadian men from 1994 to 2005 [6]. In contrast, work-related physical activity (WPA) seems to have declined over time [1, 2, 8–11]. In a Finnish cohort, the proportion of men having a physically demanding job decreased from 48 to 36% between 1982 and 2012 [2]. Little is known about physical activity trends over decades in Norway [10, 12].

The health benefits of physical activity are well documented [13–15]. Nevertheless, in most countries, less than 50% of the adult population meets the recommendations of at least 150 min weekly physical activity with moderate intensity [16, 17]. In order to develop evidence-based public health interventions, there is a need for more knowledge about correlates and determinants of physical activity [18]. Recent studies have found that high age [19–23], high body mass index (BMI) [19–21, 23], smoking [21, 24, 25], having a paid job [21], poor self-perceived health [19, 24, 26], and low socioeconomic status [27] are correlated with low physical activity, but the findings are not entirely consistent [27, 28]. While some studies report that men are more likely to be physically active in leisure time than women [23, 24, 29], other studies suggest the opposite [21, 30]. Moreover, high educational level has been associated with high [21] and low physical activity [20, 22], and with more sedentary time [26]. Finally, a recent review points at social, genetic, and physical environment, such as urban planning, transportation systems, and parks and trails, as important factors for LTPA [18]. Few studies have examined correlates of WPA [22, 31–33], and the studies present somewhat inconsistent findings. Education is the only variable that is consistently and inversely correlated with WPA, whereas findings on age, sex, and BMI varies [22, 31–33].

Previous studies vary in target populations and physical activity measurement methods, and the majority of existing studies have used a cross-sectional design when identifying correlates of physical activity. However, such studies are subject to risk of reverse causality [34], and longitudinal designs identifying determinants or predictors of physical activity may partly overcome this risk. In a previous study of the Tromsø Study population, we described longitudinal changes in LTPA, showing that LTPA tracks over time, with 50–60% of the population maintaining their LTPA level over decades, and LTPA level in early adulthood was a significant predictor for later LTPA level [35]. In this study, we examined secular trends in LTPA and WPA over the last decades in adults and elderly who participated in the Tromsø Study. Additionally, we explored potential cross-sectional correlates and longitudinal determinants of LTPA and WPA.

## Methods

### Study population

The Tromsø Study is an ongoing, community-based cohort study with repeated surveys, conducted in the municipality of Tromsø, Norway [36]. The study started in 1974 (Tromsø 1), with repeated health surveys in 1979–1980 (Tromsø 2), 1986–1987 (Tromsø 3), 1994–1995 (Tromsø 4), 2001 (Tromsø 5), and 2007–2008 (Tromsø 6). The first survey comprised men only; thereafter both women and men were invited. Total birth cohorts and/or representative samples were invited to the study.

Eligible for the WPA analyses were participants in Tromsø 2–6 who at least once answered the question about WPA (*n* = 35,737). Eligible for the LTPA were participants in Tromsø 2, 3, 5 (only those aged <70 years), and 6 who at least once answered the question about LTPA (*n* = 30,765). In Tromsø 4 and for participants aged ≥70 years in Tromsø 5, a different LTPA question was included in the questionnaire completed by these participants. Furthermore, we excluded subjects who were <20 years of age from the Tromsø 3 survey (*n* = 978), as this was the only study to include individuals aged <20 years. Altogether 29,787 subjects (LTPA) and 34,898 (WPA) aged 20 to 89 years when attending the survey(s) were included in the analyses. A total of 8621 participants had more than one measurement of LTPA. For WPA, the respective number was 8580 participants.

### Ethics approval and consent

The Tromsø Study has been approved by the Regional Norwegian Data Protection Authority and recommended by the Regional Committee of Medical and Health Research Ethics in Norway (REC North). Each participant signed a written informed consent. Consent to use the data in future research was also obtained.

### Description of study variables

In each survey, data were collected by questionnaires and physical and clinical examinations. From the questionnaires, we extracted self-reported data on current smoking (yes/no), cardiovascular disease, i.e. heart attack, stroke, and/or angina (yes/no), educational level (≤9 years, 10–12 years, and ≥13 years), self-perceived health (5 categories [4 categories in 2001]), received unemployment benefit (yes/no), and physical activity. Physical activity was assessed with separate questions on LTPA and WPA, using the “Saltin-Grimby” scale [37, 38]. LTPA was graded from 1 to 4 and the participants were instructed to choose only one of the four available options: (1) Reading, watching TV, or other sedentary activity (“*Inactive*”), (2) Walking, cycling, or other forms of exercise at least 4 h a week (“*Light physical activity*”), (3) Participation in recreational sports, heavy gardening etc. at least 4 h a week (“*Moderate physical activity*”), and (4) Participation in hard training or sports competitions regularly several times a week (“*Vigorous physical activity*”). The WPA question concerned both paid and unpaid work, and was graded from 1 to 4, using the following response options: (1) Mostly sedentary work (“*Mostly sedentary*”), (2) Work requiring a lot of walking (“*Walking*”), (3) Work requiring a lot of walking and lifting (“*Walking and lifting*”), and (4) Heavy manual labour (“*Heavy manual labour*”). Height and weight were measured to the nearest centimetre and half-kilogram (until 1995) or decimal (from 2001), with subjects wearing light clothing and no shoes. Body mass index (BMI) was calculated as weight (kg)/height^2^ (m^2^) and categorized into underweight (<18.5 kg/m^2^), healthy weight (18.5–24.99 kg/m^2^), overweight (25.0–29.99 kg/m^2^), and obese (≥30.0 kg/m^2^).

### Statistical analyses

**Fig. 1:**
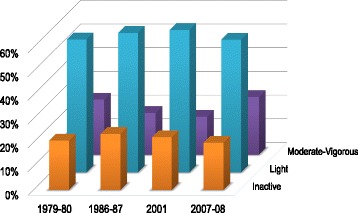
Age-standardized prevalence of LTPA 1979–2008. Secular trends in prevalence of LTPA levels (inactive, light, moderate-vigorous), adjusted for age

**Fig. 2:**
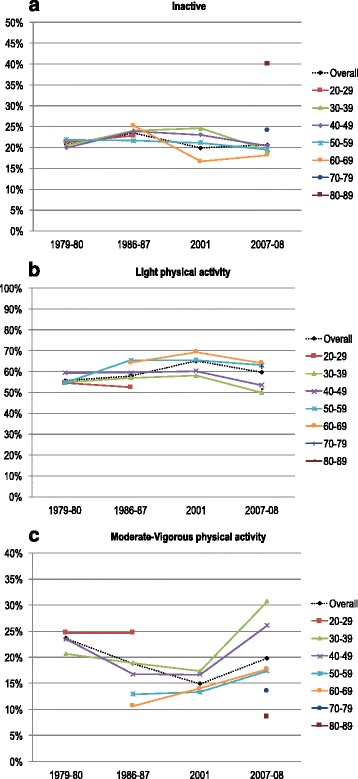
Age-specific prevalence of LTPA 1979–2008. Secular trends in prevalence of LTPA levels (inactive, light, moderate-vigorous) by age, shown as separate lines for age groups. Some age groups were not represented in all surveys

**Fig. 3:**
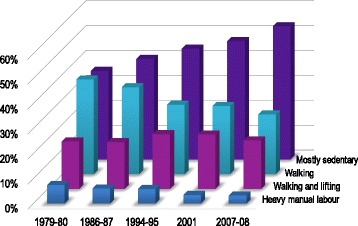
Age-standardized prevalence of WPA 1979–2008. Secular trends in prevalence of WPA levels (mostly sedentary, walking, walking and lifting, heavy manual labour), adjusted for age

Age-adjusted prevalences of LTPA and WPA were estimated using direct standardization, and secular trends over time in prevalence of LTPA and WPA were tested using generalized estimating equations (GEE) analysis (Figs. 1, 2 and 3). Identification of cross-sectional correlates of LTPA and WPA was performed for each survey separately. Potential correlates of LTPA and WPA (sex, age, education, BMI, smoking, cardiovascular disease, self-perceived health, and employment status [LTPA only]) were included in an ordinal logistic regression model using the GENLIN command in SPSS. LTPA, respectively WPA, was included in the model as an ordinal outcome variable with four categories, using the lowest physical activity level as reference group (Tables 2 and 4). Two-sided *P* <0.05 was considered statistically significant. All analyses were performed using IBM SPSS Statistics, version 23 (IBM Corporation, Armonk, NY, USA).

In a subcohort of 5044 individuals who participated in both the 1979–80 and 2007–08 surveys, we analysed the longitudinal association between possible determinants measured in 1979–80 and LTPA in 2007–08. Analyses were adjusted for sex, age, education, BMI, smoking, cardiovascular disease, and employment status in 1979–80 (Model 1, Table 3) and additionally adjusted for baseline LTPA in 1979–80 (Model 2, Table 3). Similarly, in a subcohort of 2859 participants with WPA measures in 1979–80 and 2007–09, we analysed the longitudinal association between potential determinants measured in 1979–80 (sex, age, education, BMI, smoking, cardiovascular disease, and self-perceived health) and WPA in 2007–08 (Table 5). ORs were estimated using generalized estimating equations (GEE) analysis, with LTPA, respectively WPA, in 2007–08 as an ordinal outcome variable, using the lowest physical activity category as reference group.

## Results

### Secular trends in LTPA

Participant characteristics for each survey are shown in Table 1. There were statistically significant changes in LTPA from 1979–80 to 2007–08 (age-adjusted *P* <0.001) (Fig. 1). The age-adjusted prevalence of individuals who reported being *inactive* in leisure time remained stable around 20% from 1979 to 2008 (range 19.9–23.6%). The age-adjusted prevalence of individuals engaging in *moderate* or *vigorous* LTPA decreased significantly from 23.2% in 1979–80 to 16.0% in 2001; thereafter, the prevalence increased to 24.3% in 2007–08 (*P* <0.05). These trends were seen in all age groups (Fig. 2).

**Table 1:** Participant characteristics 1979–2008

	1979-80	1986–87	1994–95	2001	2007–08
	*n* (%)	*n* (%)	*n* (%)	*n* (%)	*n* (%)
Total	16,546 (100)	20,600 (100)	20,305 (100)	5296 (100)	11,410 (100)
Sex (female)	8112 (49.0)	10,221 (49.6)	10,392 (51.2)	3002 (56.7)	5880 (51.5)
Age (years)	20–54 (men) 20–49 (women)	20–61	25–89	30–69	30–87
20–29	5597 (33.8)	5331 (25.9)	2626 (12.9)		
30–39	6263 (37.8)	6798 (33.0)	5647 (27.8)	557 (10.5)	464 (4.1)
40–49	3766 (22.8)	5120 (24.9)	5510 (27.1)	1249 (23.6)	3400 (29.8)
50–59	920 (5.6)	2966 (14.4)	3351 (16.5)	1018 (19.2)	2285 (20.0)
60–69		385 (1.9)	1259 (6.2)	2472 (46.7)	3645 (31.9)
70–79			1530 (7.5)		1349 (11.8)
80–89			382 (1.9)		267 (2.3)
LTPA					
Inactive	3427 (20.7)	4813 (23.4)		1047 (19.8)	2295 (20.1)
Light physical activity	9212 (55.7)	11,907 (57.8)		3466 (65.4)	6797 (59.6)
Moderate physical activity	3351 (20.3)	3314 (16.1)		703 (13.3)	2129 (18.7)
Vigorous physical activity	556 (3.4)	566 (2.7)		80 (1.5)	189 (1.7)
WPA					
Mostly sedentary	5813 (35.1)	8197 (39.8)	8838 (43.5)	2081 (39.3)	4428 (38.8)
Walking	6456 (39.0)	7193 (34.9)	5793 (28.5)	1228 (23.2)	2123 (18.6)
Walking and lifting	3187 (19.3)	4042 (19.6)	4344 (21.4)	848 (16.0)	1524 (13.4)
Heavy manual labour	1078 (6.5)	1151 (5.6)	1330 (6.6)	194 (3.7)	295 (2.6)
Missing	12 (0.1)	17 (0.1)		945 (17.8)	3040 (26.6)
BMI (kg/m^2^)					
< 18.5	402 (2.4)	407 (2.0)	252 (1.2)	37 (0.7)	62 (0.5)
18.5–24.9	11,370 (68.7)	13,568 (65.9)	10,752 (53.0)	2000 (37.8)	4004 (35.1)
25–29.9	3878 (23.4)	5579 (27.0)	7373 (36.3)	2273 (42.9)	5063 (44.4)
≥ 30	535 (3.2)	1020 (5.0)	1903 (9.4)	966 (18.2)	2271 (19.9)
Missing	361 (2.2)	26 (0.1)	25 (0.1)	20 (0.4)	10 (0.1)
Education level					
≤ 9 years	6048 (36.6)	6534 (31.7)	5777 (28.5)	1934 (36.5)	2911 (25.5)
10–12 years	4307 (26.0)	6186 (30.0)	7540 (37.1)	1450 (27.4)	3837 (33.6)
> 12 years	4013 (24.3)	6191 (30.1)	6934 (34.1)	1814 (34.3)	4563 (40.0)
Missing	2178 (13.2)	1689 (8.2)	54 (0.3)	98 (1.9)	99 (0.9)
Smoking					
No	8847 (51.1)	11,004 (53.4)	13,078 (64.4)	3643 (68.8)	8986 (78.8)
Yes	8098 (48.9)	9595 (46.6)	7194 (35.4)	1627 (30.7)	2286 (20.0)
Missing	1 (0.0)	1 (0.0)	33 (0.2)	27 (0.5)	138 (1.2)
Cardiovascular disease					
No	16,410 (99.2)	20,207 (98.1)	19,259 (94.8)	4749 (89.7)	10,127 (88.8)
Yes	135 (0.8)	389 (1.9)	1020 (5.0)	440 (8.3)	1060 (9.3)
Missing	2 (0.0)	4 (0.0)	26 (0.1)	107 (2.0)	223 (2.0)
Employed					
No	16,013 (96.8)	19,872 (96.5)	19,769 (97.4)	3692 (69.7)	11,356 (99.5)
Yes	519 (3.1)	722 (3.5)	536 (2.6)	57 (1.1)	54 (0.5)
Missing	14 (0.1)	6 (0.0)		1547 (29.2)	
Self-perceived health^a^					
Very bad		42 (0.2)		78 (1.5)	31 (0.3)
Bad		502 (2.4)		1553 (29.3)	501 (4.4)
Neither good nor bad		3077 (814.9)			3089 (27.1)
Good		9830 (47.7)		2977 (56.2)	5942 (52.1)
Excellent		5119 (24.8)		635 (12.0)	1776 (15.6)
Missing		2030 (9.9)		53 (1.0)	71 (0.6)

^a^In 1994–05 and 2001, the alternatives were “poor”, “not so good”, “good”, and “very good”

In general, women and men showed similar trends in LTPA over time (Additional file 1: Figure S1). Women were more likely to engage in *light* LTPA, whereas men were more likely to engage in *moderate-vigorous* LTPA. Time trends in LTPA did not differ much between age groups, nor did LTPA levels, although the highest age groups (particularly ≥80 years, only included in 2007–08) were less physically active (Fig. 2). The proportion of participants reporting being *inactive* in leisure time tended to decrease with increasing number of surveys attended (Additional file 2: Table S1).

### Secular trends in WPA

WPA changed significantly from 1979–80 to 2007–08 (*P* <0.001) (Fig. 3). The age-adjusted proportion of *mostly sedentary* WPA increased gradually between each survey, from 35.5% in 1979–80 to 53.4% in 2007–08 (*P* <0.05). This was paralleled with a significant decrease in the *walking* category from 37.9 to 23.9% (*P* <0.05). The percentage reporting *heavy manual labour* also decreased over the period, from 7.5 to 3.3% (*P* <0.05). Men were more sedentary at work than women (Additional file 3: Figure S2) (*P* <0.001). This was mainly reflected in a higher percentage of women reporting *walking* at work. A higher proportion of men than women reported *heavy manual labour*, although the prevalence decreased over time in men (*P* <0.05).

### Correlates of LTPA

In all surveys, sex, age, education, smoking, WPA, and self-perceived health were associated with LTPA (*P* <0.05), whereas cardiovascular disease and being employed were not (*P* >0.05) (Table 2). Men had higher odds of being physically active in leisure time than women in all surveys; in 2007–08, adjusted OR was 1.52 [95% CI 1.39–1.67] for men vs. women, and even higher ORs were seen in 1979–80 and 1986–87. Odds of being physically active in leisure time decreased with increasing age, although the results were not entirely consistent. Using the age group 30–39 years as reference, those aged 50–59 years had lower odds of being active in 1979–80 (adjusted OR 0.76 [95% CI 0.65–0.89], only men were included in this age group in 1979–80) and 2007–08 (adjusted OR 0.64 [95% CI 0.53–0.79]), but higher odds of being active in 1986–87 (adjusted OR 1.10 [95% CI 1.00–1.21]). Having a healthy BMI was associated with higher odds of being physically active in leisure time compared with being underweight and overweight/obese. More years of education increased the odds of being physically active in leisure time (adjusted OR 1.53 [95% CI 1.34–1.75] for >12 years of education vs. ≤9 years in 2007–08) and the time trend was stable. Smokers were less likely to be physically active than non-smokers, and similar ORs were seen in the other surveys. In all surveys, odds of being physically active in leisure time increased with increasing WPA (*P* <0.05), and those who reported excellent self-perceived health had higher odds of being active in leisure time than those who reported very bad health (adjusted OR 4.68 [95% CI 1.49–14.74] in 2007–08).

**Table 2:** Adjusted odds ratio of being at a higher LTPA level by characteristics and survey

	Adjusted Odds Ratios (95% Confidence Interval)			
	1979–80 (*n* = 14,039)	1986–87 (*n* = 18,533)	2001 (*n* = 2969)	2007–08 (*n* = 7834)
Sex				
Women	1.0	1.0	1.0	1.0
Men	2.15 (1.99–2.31)	1.87 (1.76–1.99)	1.33 (1.14–1.56)	1.52 (1.39–1.67)
Age				
20–29	1.02 (0.94–1.10)	1.17 (1.09–1.27)		
30–39	1.0	1.0	1.0	1.0
40–49	0.96 (0.88–1.04)	1.06 (0.98–1.14)	0.98 (0.79–1.23)	0.77 (0.64–0.94)
50–59	0.76 (0.65–0.89)	1.10 (1.00–1.21)	1.20 (0.94–1.53)	0.64 (0.53–0.79)
60–69		0.80 (0.65–0.99)	1.38 (1.08–1.76)	0.68 (0.56–0.84)
70–79				0.53 (0.39–0.73)
80–89				0.26 (0.13–0.53)
BMI (kg/m^2^)				
< 18.5	0.69 (0.56–0.85)	0.61 (0.49–0.75)	0.94 (0.31–2.86)	0.69 (0.35–1.35)
18.5–24.9	1.0	1.0	1.0	1.0
25–29.9	0.81 (0.76–0.89)	0.78 (0.73–0.84)	0.94 (0.79–1.11)	0.82 (0.74–0.91)
≥ 30	0.64 (0.53–0.76)	0.54 (0.48–0.62)	0.69 (0.55–0.86)	0.51 (0.45–0.58)
Education level				
≤ 9 years	1.0	1.0	1.0	1.0
10–12 years	1.19 (1.10–1.29)	1.21 (1.12–1.30)	0.97 (0.79–1.19)	1.40 (1.23–1.60)
> 12 years	1.55 (1.42–1.69)	1.45 (1.34–1.57)	1.26 (1.03–1.55)	1.53 (1.34–1.75)
Smoking				
No	1.0	1.0	1.0	1.0
Yes	0.60 (0.57–0.65)	0.57 (0.53–0.60)	0.78 (0.66–0.92)	0.53 (0.47–0.60)
Cardiovascular disease				
No	1.0	1.0	1.0	1.0
Yes	0.71 (0.49–1.03)	0.84 (0.68–1.04)	0.84 (0.59–1.21)	1.09 (0.89–1.33)
WPA				
Mostly sedentary	1.0	1.0	1.0	1.0
Walking	1.29 (1.20–1.40)	1.46 (1.37–1.56)	1.33 (1.11–1.58)	1.30 (1.17–1.45)
Walking and lifting	1.50 (1.36–1.65)	1.69 (1.56–1.84)	1.42 (1.16–1.74)	1.28 (1.13–1.45)
Heavy manual labour	1.54 (1.33–1.79)	1.98 (1.73–2.28)	2.61 (1.68–4.05)	1.56 (1.29–2.02)
Employed				
No	1.0	1.0	1.0	
Yes	0.82 (0.67–0.99)	0.90 (0.77–1.05)	1.58 (0.82–3.06)	
Self-perceived health^a^				
Very bad		1.0	1.0	1.0
Bad		0.82 (0.44–1.54)	1.89 (0.73–4.90)	0.85 (0.26–2.73)
Neither good nor bad		0.89 (0.48–1.64)		1.18 (0.38–3.69)
Good		1.40 (0.76–2.58)	2.80 (1.09–7.20)	2.18 (0.70–6.84)
Excellent		2.28 (1.24–4.21)	5.78 (2.21–15.11)	4.68 (1.49–14.74)

Adjusted for sex, age, BMI, smoking, cardiovascular disease, WPA, education level, employment status, and self-perceived health in each survey

^a^In 2001, the alternatives were “poor”, “not so good”, “good”, and “very good”

Longitudinal associations between participant characteristics in 1979–80 and LTPA in 2007–08 showed similar results as the cross-sectional associations, also after adjustment for LTPA level at baseline in 1979–80 (Table 3). Sex, age, education, BMI, smoking, and WPA in 1979–80 were significant determinants of LTPA 28 years later (*P* <0.05). Being physically active in leisure time in 1979–80 were highly associated with being in a higher LTPA category in 2007–08 (*P* <0.05).

**Table 3:** Adjusted odds ratio of being at a higher LTPA level in 2007–08 by characteristics in 1979–80

	Adjusted Odds Ratios (95% Confidence Interval)	
	Model 1^a^ (*n* = 5044)	Model 2^b^ (*n* = 5037)
Sex		
Women	1.0	1.0
Men	1.77 (1.56–2.02)	1.47 (1.28–1.67)
Age		
20–29 years	1.04 (0.89–1.21)	1.06 (0.91–1.24)
30–39 years	1.0	1.0
40–49 years	0.94 (0.82–1.07)	0.93 (0.81–1.05)
50–54 years	0.66 (0.45–0.98)	0.72 (0.49–1.03)
BMI (kg/m^2^)		
< 18.5	0.91 (0.61–1.38)	0.92 (0.61–1.39)
18.5–24.9	1.0	1.0
25–29.9	0.77 (0.67–0.89)	0.83 (0.72–0.96)
≥ 30	0.42 (0.29–0.59)	0.48 (0.34–0.68)
Education level		
≤ 9 years	1.0	1.0
10–12 years	1.22 (1.06–1.40)	1.17 (1.02–1.34)
> 12 years	1.37 (1.18–1.60)	1.26 (1.08–1.47)
Smoking		
No	1.0	1.0
Yes	0.64 (0.57–0.72)	0.71 (0.63–0.80)
Self-reported cardiovascular disease		
No	1.0	1.0
Yes	0.44 (0.18–1.05)	0.40 (0.15–1.03)
WPA		
Mostly sedentary	1.0	1.0
Walking	1.17 (1.03–1.34)	1.10 (0.96–1.26)
Walking and lifting	1.11 (0.93–1.32)	0.99 (0.83–1.18)
Heavy manual labour	1.55 (1.18–2.05)	1.41 (1.06–1.87)
Employed		
No	1.0	1.0
Yes	0.96 (0.65–1.41)	1.02 (0.69–1.52)
LTPA		
Inactive		1.0
Light		2.58 (2.20–3.02)
Moderate		4.85 (3.96–5.94)
Vigorous		10.78 (7.47–15.56)

Age 20–54 years (men) or 20–49 years (women) at baseline

^a^Model 1: Adjusted for sex, age, BMI, smoking, cardiovascular disease, WPA, education level, and employment status in 1979–80

^b^Model 2: Model 1 + additional adjustment for baseline LTPA in 1979–80

### Correlates of WPA

Sex, age, education, smoking, and LTPA were associated with WPA in most surveys (Table 4). Odds of being in a higher level of WPA decreased significantly with increasing age in all surveys except 2007–08, and with increasing education level (*P* <0.05). Association between sex and WPA shifted during the time period, from men being more likely to have a physically demanding work in 1979–89 (adjusted OR 1.31 [95% CI 1.22–1.41] for men vs. women) to women having higher odds of high WPA in 2007–08 (adjusted OR 0.90 [95% CI 0.82–0.98] for men vs. women). Daily smokers were 11–24% more likely to have physically demanding work (*P* <0.05). BMI and cardiovascular disease were not significantly associated with WPA (*P* >0.05).

**Table 4:** Adjusted odds ratio of being at a higher WPA level by characteristics and survey

	Adjusted Odds Ratios (95% Confidence Interval)				
	1979–80 (*n* = 14,045)	1986–87 (*n* = 18,535)	1994–95 (*n* = 20,066)	2001 (*n* = 3567)	2007–08 (*n* = 7834)
Sex					
Women	1.0	1.0	1.0	1.0	1.0
Men	1.31 (1.22, 1.41)	1.22 (1.16, 1.30)	1.05 (0.99, 1.11)	0.92 (0.81, 1.06)	0.90 (0.82, 0.98)
Age					
20–29	1.34 (1.24, 1.44)	1.44 (1.34, 1.55)	1.24 (1.13, 1.35)		
30–39	1.0	1.0	1.0	1.0	1.0
40–49	0.91 (0.83, 0.98)	0.73 (0.68, 0.79)	0.77 (0.72, 0.83)	0.74 (0.60, 0.90)	0.93 (0.77, 1.14)
50–59	0.74 (0.64, 0.87)	0.75 (0.69, 0.83)	0.65 (0.60, 0.71)	0.55 (0.44, 0.69)	0.81 (0.66, 1.00)
60–69		0.66 (0.53, 0.81)	0.77 (0.68, 0.87)	0.50 (0.41, 0.63)	0.71 (0.58, 0.87)
70–79			1.12 (1.00–1.26)		
80–89^a^					
BMI (kg/m^2^)					
< 18.5	1.03 (0.85, 1.26)	0.99 (0.81, 1.21)	0.82 (0.64, 1.03)	1.14 (0.50, 2.59)	1.37 (0.75, 2.50)
18.5–24.9	1.0	1.0	1.0	1.0	1.0
25–29.9	1.04 (0.96, 1.12)	1.06 (0.99, 1.13)	1.00 (0.95, 1.06)	1.04 (0.90, 1.20)	0.96 (0.87, 1.06)
≥ 30	1.18 (0.99, 1.40)	1.05 (0.92, 1.20)	1.01 (0.92, 1.11)	0.85 (0.70, 1.03)	0.95 (0.83, 1.08)
Education level					
≤ 9 years	1.0	1.0	1.0	1.0	1.0
10–12 years	0.39 (0.36, 0.43)	0.43 (0.40, 0.46)	0.42 (0.39, 0.44)	0.55 (0.47, 0.65)	0.53 (0.47, 0.60)
> 12 years	0.19 (0.18, 0.21)	0.23 (0.21, 0.25)	0.20 (0.18, 0.21)	0.28 (0.23, 0.33)	0.21 (0.18, 0.23)
Smoking					
No	1.0	1.0	1.0	1.0	1.0
Yes	1.15 (1.08, 1.23)	1.14 (1.08, 1.21)	1.11 (1.05, 1.18)	1.13 (0.98, 1.30)	1.24 (1.11, 1.39)
Cardiovascular disease					
No	1.0	1.0	1.0	1.0	1.0
Yes	0.71 (0.49, 1.02)	0.62 (0.51, 0.77)	1.03 (0.91, 1.18)	0.79 (0.60, 1.05)	1.02 (0.84, 1.25)
LTPA^b^					
Inactive	1.0	1.0	1.0	1.0	1.0
Light	1.43 (1.32, 1.55)	1.50 (1.40, 1.60)	0.97 (0.91, 1.05)	1.55 (1.32, 1.83)	1.36 (1.21, 1.53)
Moderate	1.64 (1.48, 1.82)	2.01 (1.83, 2.20)	1.00 (0.93, 1.07)	1.94 (1.55, 2.42)	1.53 (1.32, 1.77)
Vigorous	1.32 (1.08, 1.60)	1.86 (1.56, 2.23)	1.42 (1.29, 1.55)	1.73 (1.07, 2.81)	1.33 (0.95, 1.85)
Self-perceived health^c^					
Very bad		1.0		1.0	1.0
Bad		1.25 (0.68, 2.33)		1.19 (0.60, 2.36)	2.07 (0.58, 7.39)
Neither good nor bad		2.24 (1.23, 4.09)			2.98 (0.86, 10.37)
Good		2.24 (1.23, 4.08)		1.06 (0.54, 2.10)	2.77 (0.80, 9.65)
Excellent		2.10 (1.15, 3.82)		0.92 (0.46, 1.86)	2.55 (0.73, 8.92)

Adjusted for sex, age, BMI, smoking, cardiovascular disease, LTPA, education level, and self-perceived health

^a^Few people reported WPA

^b^In 1994–95, a different LTPA was used (Hard physical activity 0, <1, 1–2, ≥3 h/week)

^c^In 2001, the alternatives were “poor”, “not so good”, “good”, and “very good”

In the longitudinal models, only education level and WPA measured in 1979–80 showed an association with WPA in 2007–08, even after adjustment for WPA level at baseline (Table 5). Odds of being in a high level of WPA in 2007–08 decreases significantly with education level in 1979–80, and high WPA in 1979–80 was a strong predictor for being physically active at work in 2007–08 (*P* <0.05).

**Table 5:** Adjusted odds ratio of being at a higher WPA level in 2007–08 by characteristics in 1979–80

	Adjusted Odds Ratios (95% Confidence Interval)	
	Model 1^a^ (*n* = 2859)	Model 2^b^ (*n* = 2854)
Sex		
Women	1.0	1.0
Men	1.05 (0.89, 1.23)	0.98 (0.83, 1.17)
Age		
20–29	1.25 (1.06, 1.48)	1.12 (0.94, 1.33)
30–39	1.0	1.0
40–49	1.16 (0.92, 1.46)	1.14 (0.89, 1.46)
50–54	0.66 (0.34, 1.29)	0.56 (0.27, 1.18)
BMI (kg/m^2^)		
< 18.5	0.87 (0.56, 1.36)	0.95 (0.61, 1.47)
18.5–24.9	1.0	1.0
25–29.9	1.10 (0.92, 1.32)	1.15 (0.95, 1.39)
≥ 30	1.17 (0.73, 1.88)	1.08 (0.65, 1.77)
Education level		
≤ 9 years	1.0	1.0
10–12 years	0.45 (0.38, 0.53)	0.63 (0.52, 0.75)
> 12 years	0.24 (0.20, 0.29)	0.41 (0.33, 0.50)
Smoking		
No	1.0	1.0
Yes	0.93 (0.80, 1.08)	0.93 (0.79, 1.09)
Self-reported cardiovascular disease		
No	1.0	1.0
Yes	1.12 (0.13, 9.38)	1.38 (0.12, 15.22)
LTPA		
Inactive		1.0
Light	1.29 (1.06, 1.56)	1.06 (0.86, 1.29)
Moderate	1.39 (1.11, 1.75)	1.09 (0.86, 1.39)
Vigorous	0.93 (0.63, 1.38)	0.87 (0.58, 1.31)
WPA		
Mostly sedentary	1.0	1.0
Walking		4.59 (3.78, 5.56)
Walking and lifting		8.87 (6.99, 11.26)
Heavy manual labour		18.03 (12.38, 26.25)

Age 20–54 years (men) or 20–49 years (women) at baseline

^a^Model 1: Adjusted for sex, age, BMI, smoking, cardiovascular disease, LTPA, and education level in 1979–80

^b^Model 2: Model 1 + additional adjustment for baseline WPA in 1979–80

## Discussion

A main finding of this study is that between 1979 and 2008, this cohort of Norwegian adults and elderly became more sedentary at work, whereas the proportion of inactive in leisure time remained fairly stable. The proportion of individuals performing moderate-vigorous LTPA showed a U-shape over time, with an increase from 2001 to 2007–08. Altogether, our study suggests that the overall physical activity level has declined from 1979 to 2008. Another main finding is that several individual-level factors were associated with LTPA and WPA. Sex, age, education, and smoking were significant cross-sectional correlates of both LTPA and WPA, although partly in different directions. Cardiovascular disease and being employed were the only variables included in the model that did not correlate with LTPA, whereas cardiovascular disease and BMI were not correlated with WPA. The associations between correlates and LTPA were consistent across surveys and confirmed in longitudinal analyses, which showed that sex, age, education, smoking, WPA, and LTPA were determinants of LTPA measured 28 years later. In contrast, most cross-sectional associations with WPA disappeared in the longitudinal models.

### Secular trends in LTPA and WPA

Despite the evident changes towards a more sedentary lifestyle seen in most Western societies, studies on LTPA are remarkably consistent in showing an increase in LTPA over the last decades [1–9]. Although WPA seems to have declined [1, 2, 8–11], the LTPA findings suggest that there is no apparent decline in total physical activity in many Western societies. One explanation for these somewhat surprising findings may be lack of adequate instruments to capture all aspects of physical activity during the day, such as light-intensity activity like housework, gardening etc. Over the last decades, physical activity has predominantly been measured by questionnaires, and only recently, objective instruments such as accelerometers have been introduced. In our cohort, LTPA was fairly stable from 1979 to 2008, whereas other studies [1–9] have found an increase over the same time period, suggesting that our cohort may have a more modest development in LTPA. As in most comparable studies, we found that WPA decreased over time [1, 2, 8–11]. However, comparison between studies is impeded by use of different definitions, questions, and categories of physical activity.

### Correlates of LTPA

Knowledge of correlates and determinants of physical activity is important for public health promotions. In accordance with most previous studies, the present study identified sex, age, education, and smoking as major correlates and determinants of LTPA. In line with our findings, most previous research have shown that age is inversely associated with activity level [19–21, 24, 29, 39, 40]. Furthermore, we found that men were more likely to be physically active in leisure time than women, in agreement with some previous studies [23, 24, 29]. In contrast, other studies have found that women were more likely to be physically active than men [21, 30]; however, these studies tended to involve older individuals. Smokers were less likely to be physically active in leisure time than non-smokers, a finding that is consistent with other studies [21, 24, 25].

In the present study, we observed a U-shaped association between BMI and LTPA, in that those with healthy BMI were more likely to be physically active than those who were underweight or overweight. This finding is in contrast with previous studies, which have shown an inverse, linear association between BMI and physical activity [19–21, 23, 26]. However, previous studies have analysed BMI on a continuous scale [21, 26] or did not examine underweight as a separate category [20, 23, 24]. Interestingly, one of the few studies that incorporated underweight (<18.5 kg/m^2^) as a separate category found results similar to this study, in that the odds of being physically active were lower among underweight and obese individuals, compared with healthy weight individuals [40]. Our longitudinal analyses showed a weaker relationship between underweight and LTPA, and future research is warranted to elucidate whether underweight is a determinant of low LTPA.

One finding of our study was that more years of education increased the odds of being physically active in leisure time. This finding is supported by some [21, 40, 41], but not all previous studies [20]. In a study from Cameroon, Assah and colleagues found that high education level was associated with lower LTPA [20]. These contrasting findings may indicate that local culture is a predictor for engagement in LTPA. We did not find any association between being employed and LTPA in the present study, in contrast to previous studies, which have observed that having a paid job is associated with low LTPA [19, 21, 29]. We found a positive association between WPA and LTPA, in accordance with Solomon et al. [23] and Macera et al. [40]. Not surprisingly, the longitudinal analyses revealed that baseline LTPA was a strong determinant of LTPA later in life, as we have shown in a previous study [35].

Several studies have found an association between poor self-perceived health and low physical activity [19, 24, 26]. Consistent with these studies, our study showed that the odds of engaging in LTPA increased positively as self-perceived health improved. These findings accentuate the pitfall of reverse causality in studies of associations between physical activity and health outcomes; physically active individuals may be healthy because of the activity, or they may engage in physical activity because they are healthy. As studies vary largely in demographics, design, and physical activity domain and classification, divergent findings related to correlates of physical activity may partly be ascribed to methodological differences.

### Correlates of WPA

Correlates of WPA were in essence consistent with correlates of LTPA. However, some correlates, such as smoking and education, related to WPA and LTPA differently, suggesting that smokers or individuals with low education level, who were inactive in leisure time, to some degree may compensate with higher WPA level. Although the cross-sectional correlates of WPA were consistent across surveys, most associations disappeared in the longitudinal analyses, which calls for further longitudinal studies on correlates of WPA. Few studies have examined correlates of WPA, and education is the only variable in previous studies that is consistently correlated with WPA [22, 31–33], showing an inverse relation to WPA in accordance with this study. One study showed that age was inversely correlated with WPA [22], similar to our findings, whereas other studies did not find any correlation between age and WPA [31, 33]. Previous studies did not find any associations between sex and WPA [22, 31, 33], in contrast to our study; however, our findings disclosed that the association with sex shifted over time.

### Limitations and strengths

Our study has some limitations. Even with high participation rates in all surveys (65–78%), nonparticipants may differ from participants, introducing potential selection bias. Studies of nonparticipants show that women and older individuals are more likely to participate in population-based health studies [36], and nonparticipants are shown to be more prevalent smokers and more likely single [42], to have lower socioeconomic status, poorer health and higher mortality than participants [43, 44], although this may depend on the disorder or diagnose of interest [43, 44].

The “Saltin-Grimby” LTPA and WPA questions [37, 38] utilized in this study have been used in many other population studies [12, 45–48] and thoroughly validated against accelerometer and fitness measures [37, 45, 49–51]. A recent validation study showed that the “Saltin-Grimby” LTPA question was positively associated with objectively measured physical activity (accelerometer) and maximal oxygen uptake in a dose–response relationship [51]. However, the participant substantially overestimated their physical activity level when reporting through the “Saltin-Grimby” question compared with the accelerometer measurements [51], indicating that the prevalences observed in the present study most likely are an overestimate of the true physical activity level and may not be taken as exact magnitudes.

The strengths of this study is the large cohort, the long time period and high participation rates, enabling high representability, which is imperative when studying prevalences. Furthermore, we were able to utilize a longitudinal design in a subsample of more than 5000 participants.

## Conclusion

This cohort study of adults and elderly showed that physical activity levels declined from 1979 to 2008. The proportion of inactive in leisure time remained stable, suggesting that the decline in WPA is the main cause for the decline in total physical activity level. The most inactive individuals in leisure time were female, older, smokers, obese or underweight, and had lower education, which suggest that public physical activity interventions want to consider aiming at these target groups. We call for further research that include social and occupational factors, and physical environment as potential facilitators of physical activity.

## Additional files

Additional file 1: Figure S1. Secular trends in sex-specific prevalence of LTPA 1979–2008. (PDF 28 kb)
Additional file 2: Table S1. Characteristics of participants by number of surveys attended. (DOCX 17 kb)
Additional file 3: Figure S2. Secular trends in sex-specific prevalence of WPA 1979–2008. (PDF 32 kb)

## Abbreviations

BMI: Body mass index
CI: Confidence interval
LTPA: Leisure time physical activity
OR: Odds ratio
WPA: Work-related physical activity
