# Supplementary figures and images for: Secular trends and correlates of physical activity: The Tromsø Study 1979-2008
Source: BMC Public Health. 2016 Dec 3;16:1215. doi: 10.1186/s12889-016-3886-z (PMC5135806; doi:10.1186/s12889-016-3886-z)

### a. Inactive

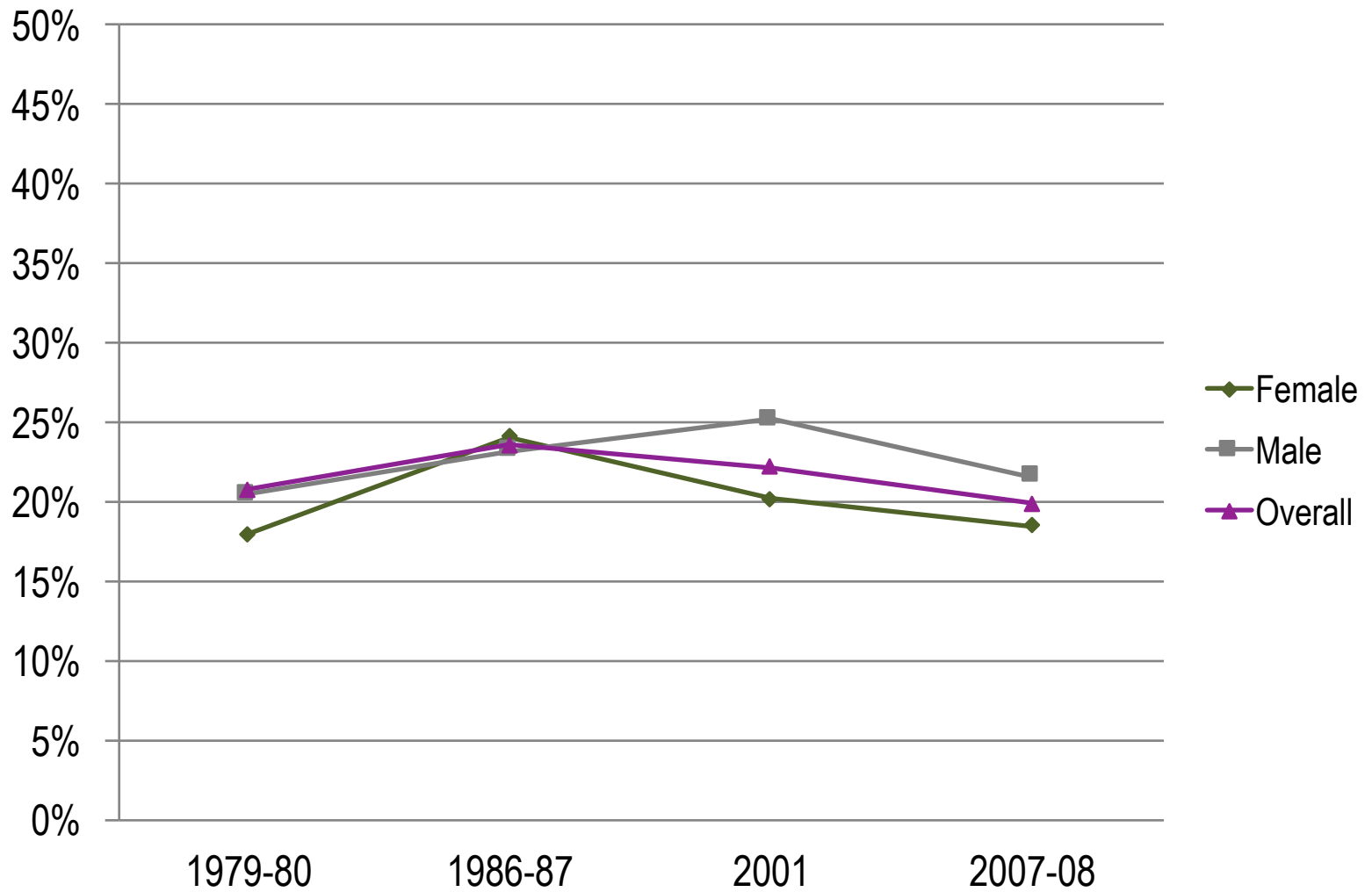

### b. Light activity

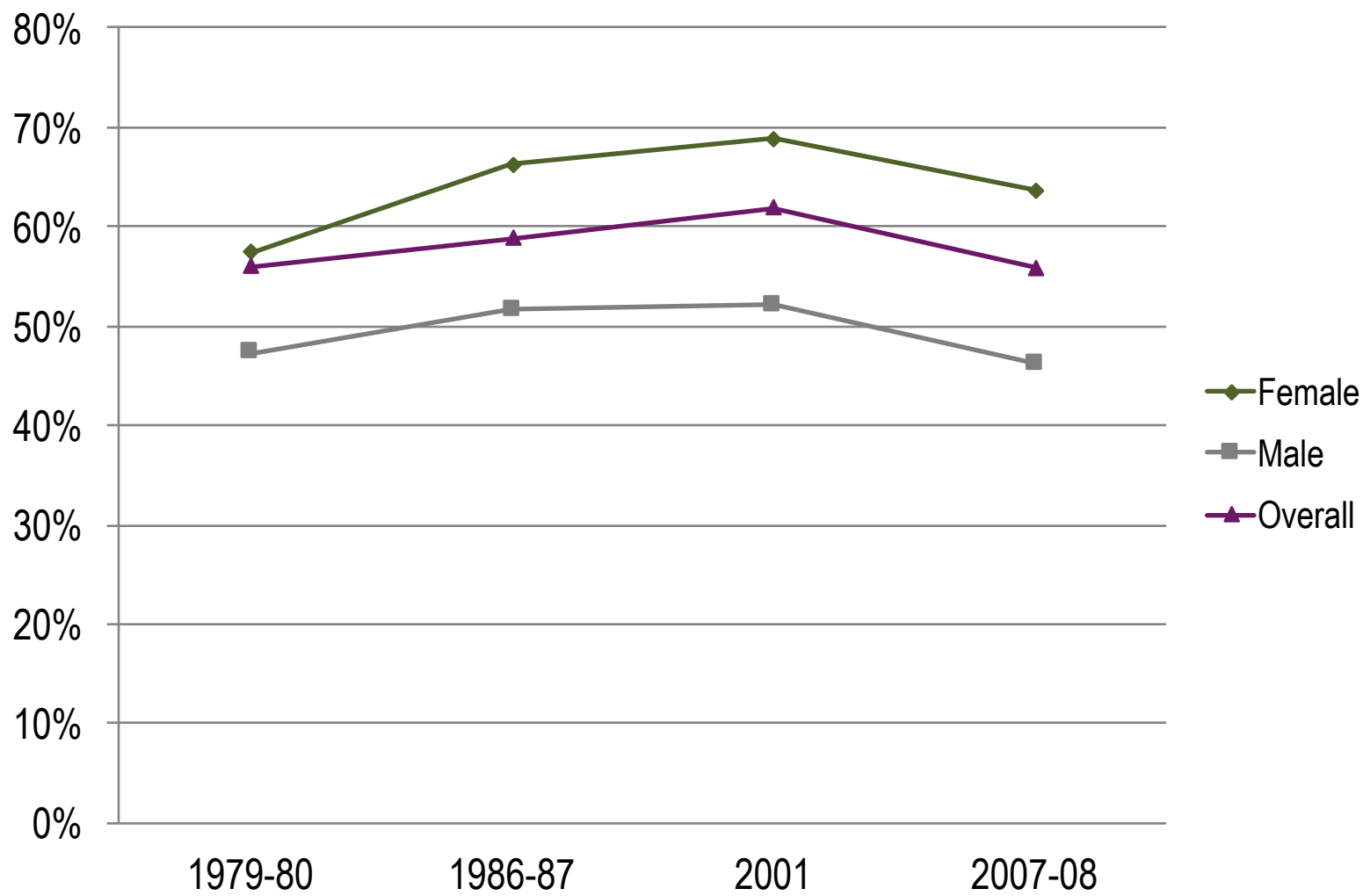

### c. Moderate-Vigorous activity

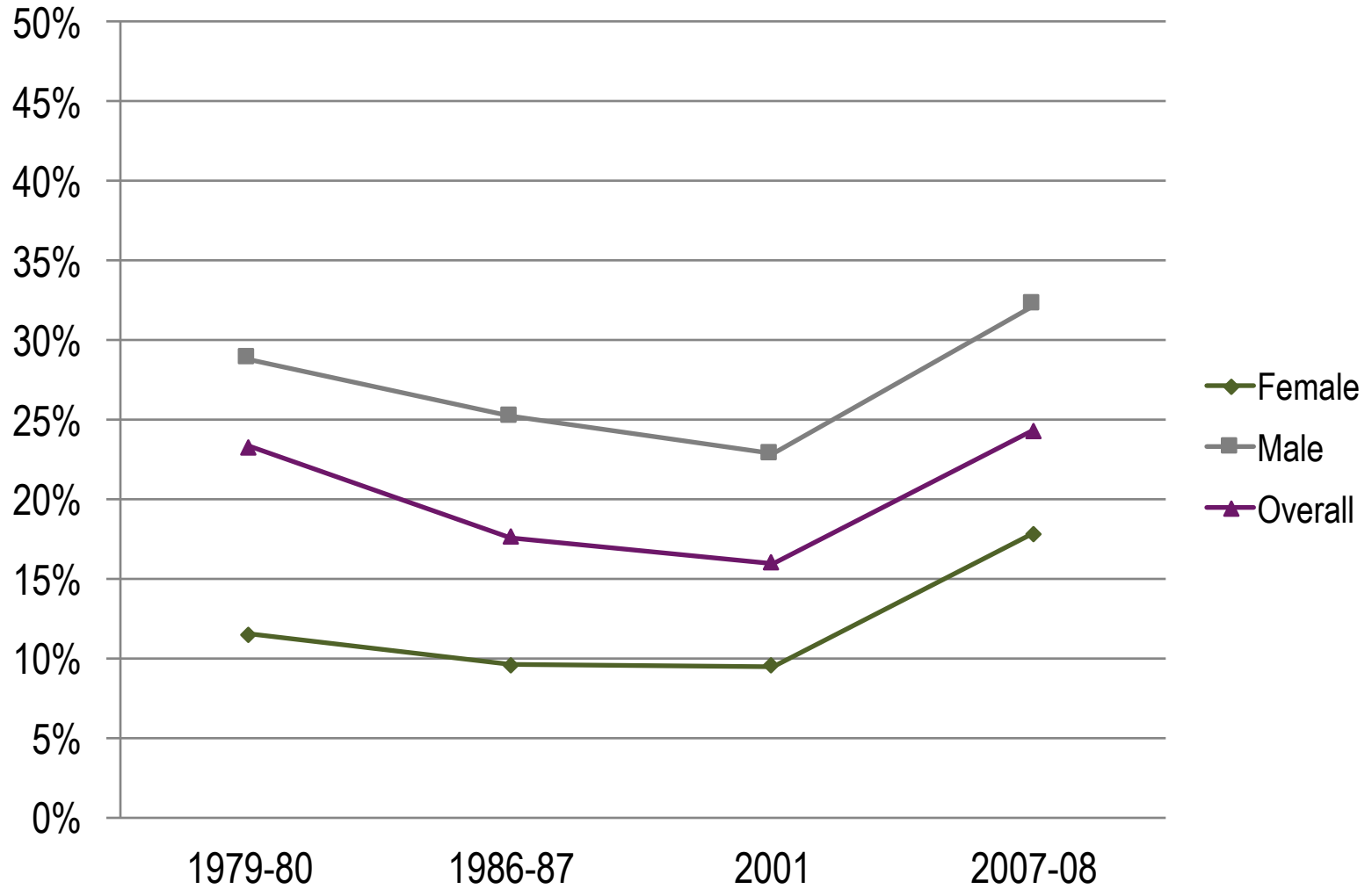

Supplement: Additional file 1: Figure S1. — Secular trends in sex-specific prevalence of LTPA 1979–2008. (PDF 28 kb) [file 12889_2016_3886_MOESM1_ESM.pdf]

### a. Mostly sedentary

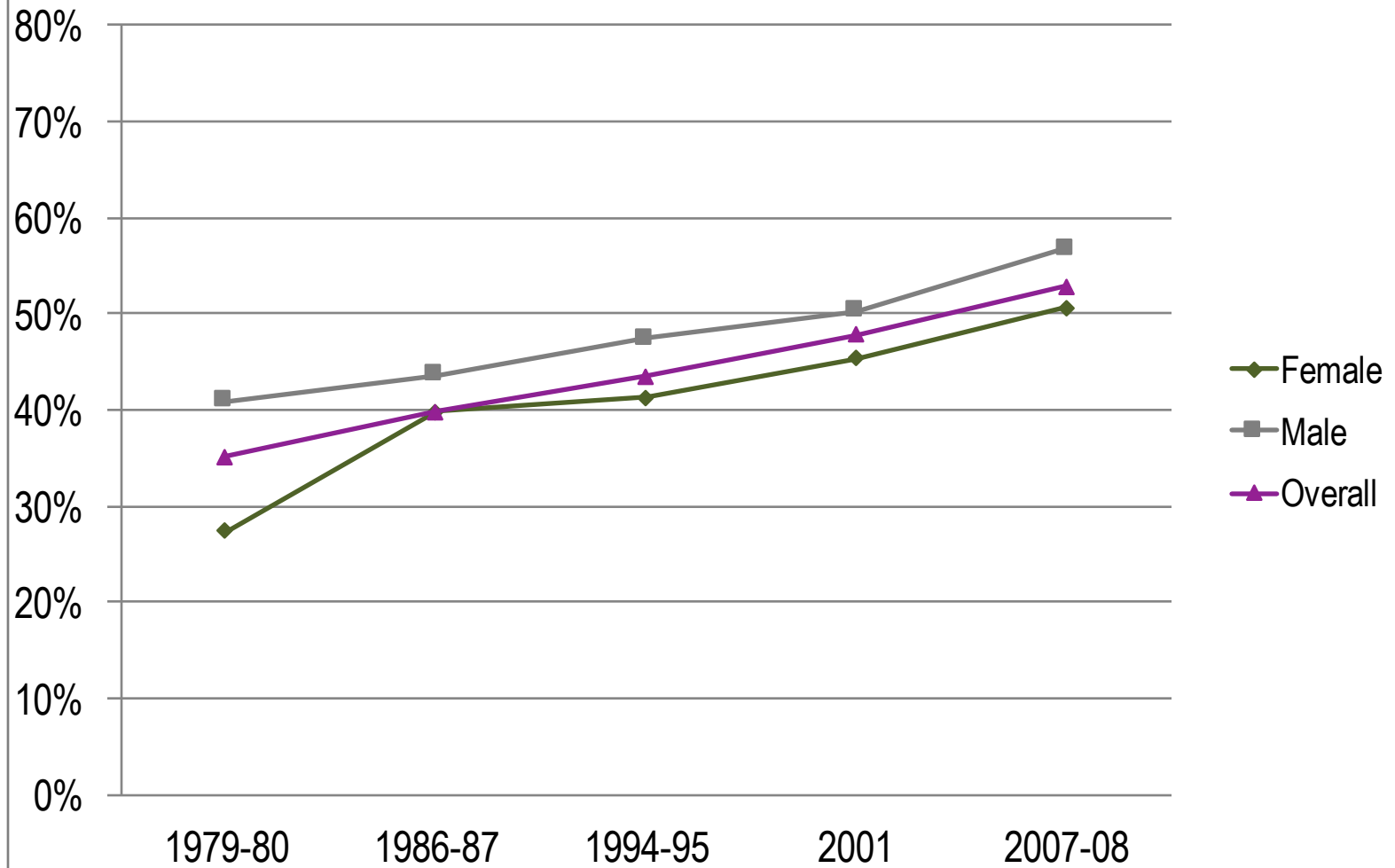

## b. Walking

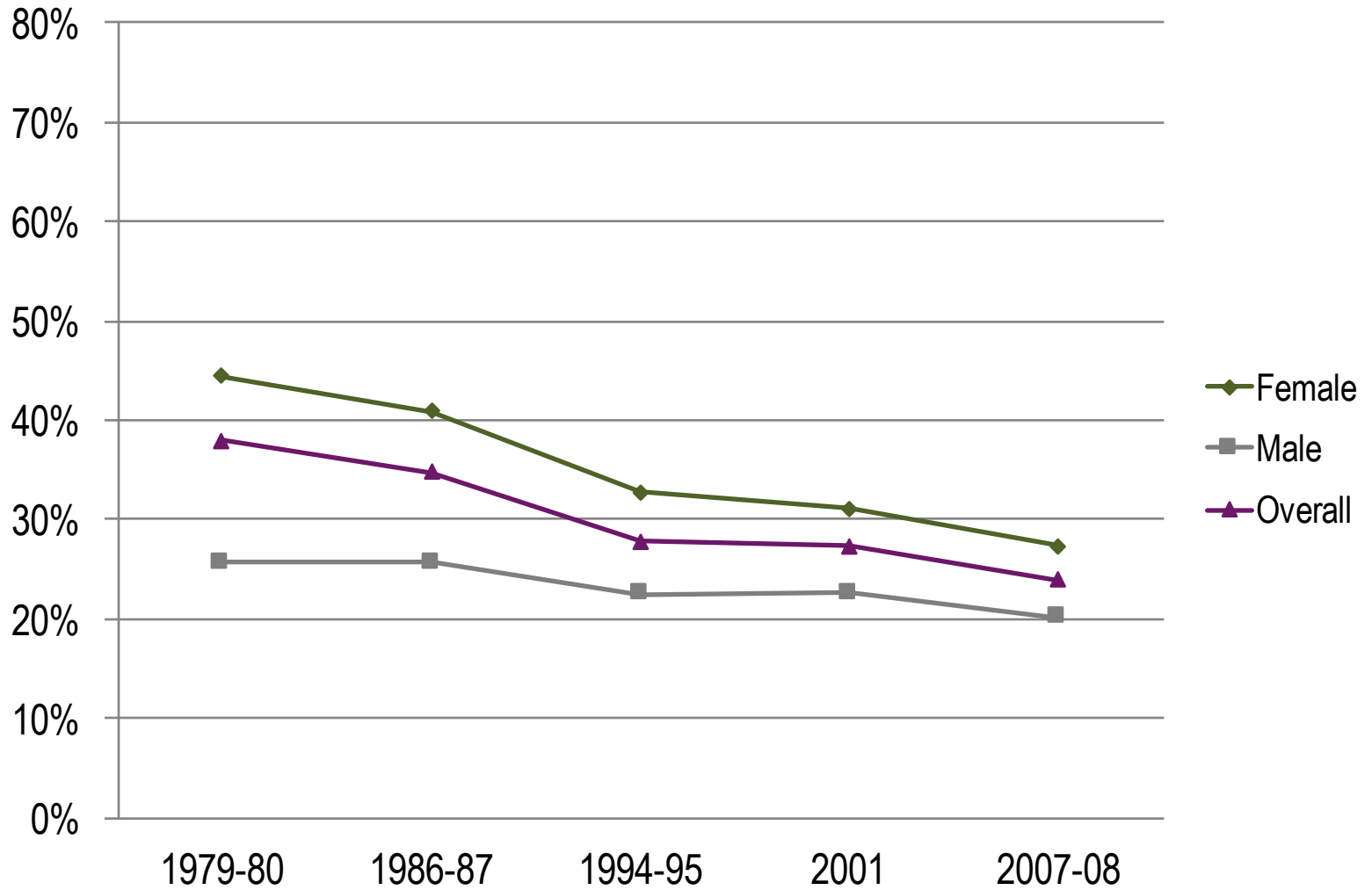

### c. Walking and lifting

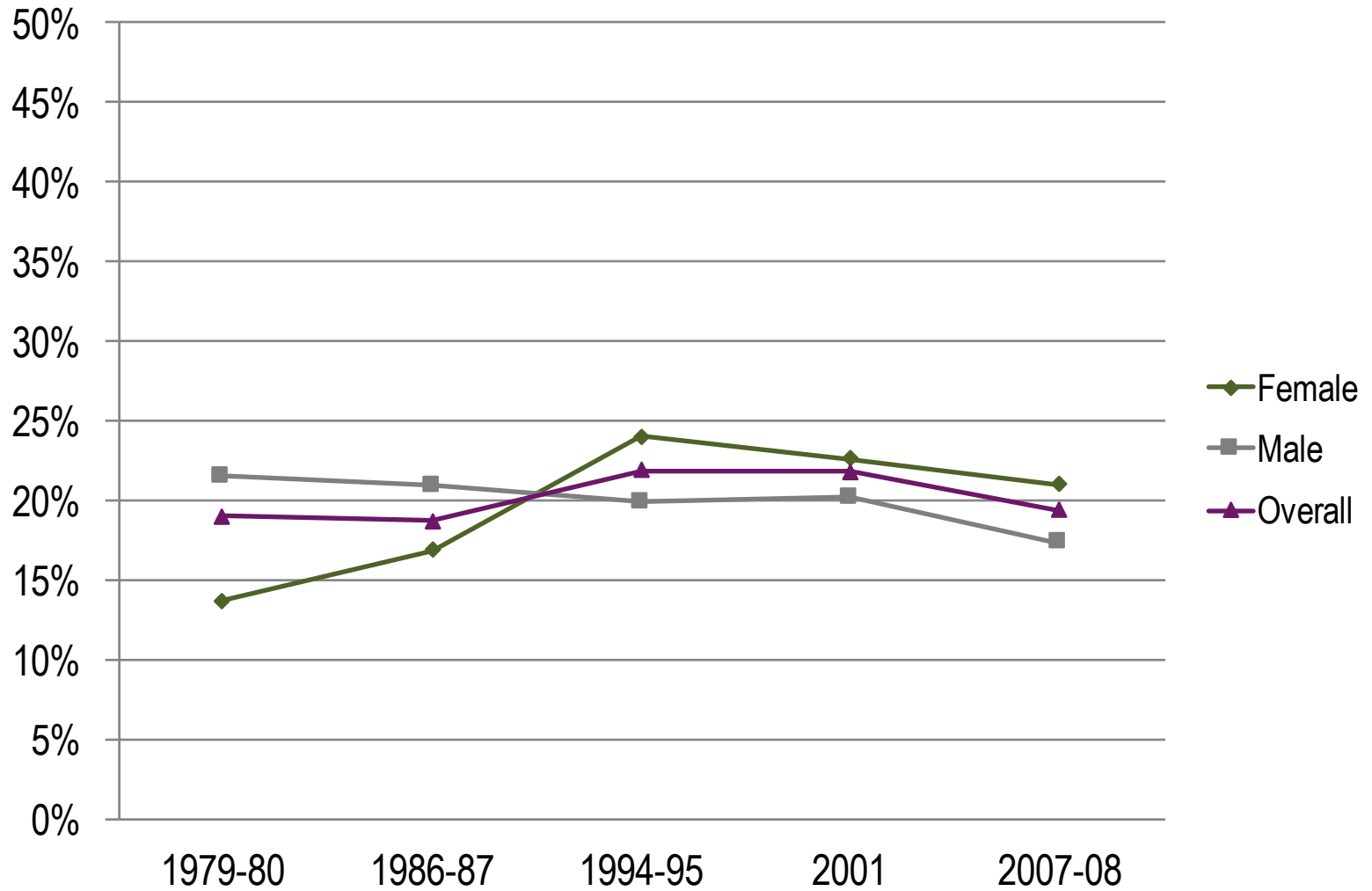

#### d. Heavy manual labor

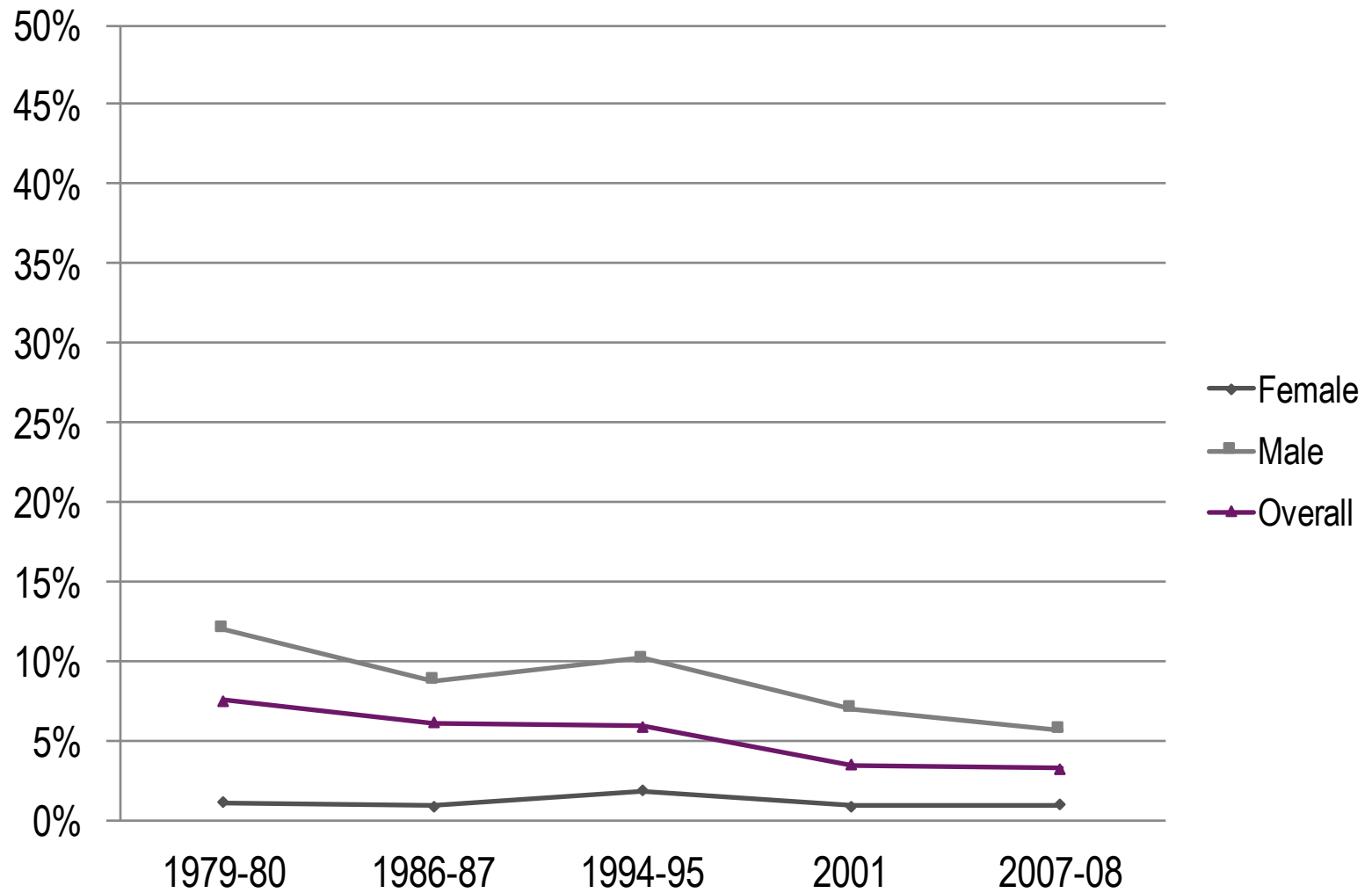

Supplement: Additional file 3: Figure S2. — Secular trends in sex-specific prevalence of WPA 1979–2008. (PDF 32 kb) [file 12889_2016_3886_MOESM3_ESM.pdf]
