# Supplementary material for: Secular trends and correlates of physical activity: The Tromsø Study 1979-2008
Source: BMC Public Health. 2016 Dec 3;16:1215. doi: 10.1186/s12889-016-3886-z (PMC5135806; doi:10.1186/s12889-016-3886-z)
Supplement: Additional file 2: Table S1. — Characteristics of participants by number of surveys attended. (DOCX 17 kb) [file 12889_2016_3886_MOESM2_ESM.docx]

Table S1: Characteristics of participants by number of surveys attended

|  | Number of surveys attended | | | |
| --- | --- | --- | --- | --- |
|  | 1 survey | 2 surveys | 3 surveys | 4 surveys |
| Total n | 13572 | 8617 | 4621 | 2296 |
| LTPA 1979-80 |  |  |  |  |
| n | 3895 | 6145 | 4211 | 2296 |
| Inactive | 794 (20.4) | 1392 (22.7) | 818 (19.4) | 423 (18.4) |
| Light physical activity | 2187 (56.1) | 3364 (54.7) | 2342 (55.6) | 1320 (57.5) |
| Moderate physical activity | 772 (19.8) | 1173 (19.1) | 906 (21.5) | 500 (21.8) |
| Vigorous physical activity | 142 (3.6) | 216 (3.5) | 145 (3.4) | 53 (2.3) |
| LTPA 1986-87 |  |  |  |  |
| n | 5769 | 7974 | 4561 | 2296 |
| Inactive | 1403 (24.3) | 1937 (24.3) | 997 (21.9) | 476 (20.7) |
| Light physical activity | 3142 (54.5) | 4571 (57.3) | 2736 (60.0) | 1458 (63.5) |
| Moderate physical activity | 959 (16.6) | 1259 (15.8) | 755 (16.6) | 341 (14.9) |
| Vigorous physical activity | 265 (4.6) | 207 (2.6) | 73 (1.6) | 21 (0.9) |
|  |  |  |  |  |
| LTPA 2001 |  |  |  |  |
| n | 745 | 769 | 1486 | 2296 |
| Inactive | 185 (24.8) | 165 (21.5) | 318 (21.4) | 379 (16.5) |
| Light physical activity | 429 (57.6) | 478 (62.2) | 960 (64.6) | 1599 (69.6) |
| Moderate physical activity | 100 (13.4) | 109 (14.2) | 189 (12.7) | 305 (13.3) |
| Vigorous physical activity | 31 (4.2) | 17 (2.2) | 19 (1.3) | 13 (0.6) |
|  |  |  |  |  |
| LTPA 2007-08 |  |  |  |  |
| n | 3163 | 2346 | 3605 | 2296 |
| Inactive | 698 (22.1) | 505 (21.5) | 707 (19.6) | 385 (16.8) |
| Light physical activity | 1681 (53.1) | 1325 (56.5) | 2250 (62.4) | 1541 (67.1) |
| Moderate physical activity | 687 (21.7) | 475 (20.2) | 609 (16.9) | 358 (15.6) |
| Vigorous physical activity | 97 (3.1) | 41 (1.7) | 39 (1.1) | 12 (0.5) |
